# Carotenoid composition and conformation in retinal oil droplets of the domestic chicken*

**DOI:** 10.1371/journal.pone.0217418

**Published:** 2019-05-31

**Authors:** Ana-Andreea Arteni, Amy M. LaFountain, Maxime T. A. Alexandre, Mathias Fradot, Maria M. Mendes-Pinto, José-Alain Sahel, Serge Picaud, Harry A. Frank, Bruno Robert, Andrew A. Pascal

**Affiliations:** 1 Institute for Integrative Biology of the Cell (I2BC), CEA, CNRS, Université Paris-Saclay, Gif-sur-Yvette, France; 2 Department of Chemistry, University of Connecticut, Storrs, Connecticut, United States of America; 3 Sorbonne University, INSERM, CNRS, Institut de la Vision, Paris, France; University of Florida, UNITED STATES

## Abstract

Carotenoid-containing oil droplets in the avian retina act as cut-off filters to enhance colour discrimination. We report a confocal resonance Raman investigation of the oil droplets of the domestic chicken, *Gallus gallus domesticus*. We show that all carotenoids present are in a constrained conformation, implying a locus in specific lipid binding sites. In addition, we provide proof of a recent conclusion that all carotenoid-containing droplets contain a mixture of all carotenoids present, rather than only a subset of them—a conclusion that diverges from the previously-held view. Our results have implications for the mechanism(s) giving rise to these carotenoid mixtures in the differently-coloured droplets.

## Introduction

Colour vision in birds is highly developed, and is even more sophisticated than that of humans and other primates. In common with a number of other species (including some fish and reptiles), their visual colour system is tetrachromatic, due to the presence of four types of cone photoreceptor—categorised into long-, medium-, and two types of short-wavelength-sensitive forms (LWS, MWS, SWS2 & SWS1, respectively; [1]). In certain species, the SWS1 opsin is tuned to even shorter wavelengths to extend this visual system into the UV. Further complexity is seen through the presence of double cones, which contain LWS photoreceptors and are thought to be responsible for luminance and/or motion detection [1]. Colour distinction between the photoreceptor types is further refined by the presence of coloured oil droplets. These highly-refractile, spherical organelles are located anterior to the visual pigment, such that incident light passes through them first. The range of colours displayed by oil droplets, due to the presence of specific carotenoid molecules, thus allows them to act as chromatic cut-off filters, enhancing colour discrimination. The evolution and function of oil droplets have been the subject of two recent reviews [2,3].

**Fig 1:**
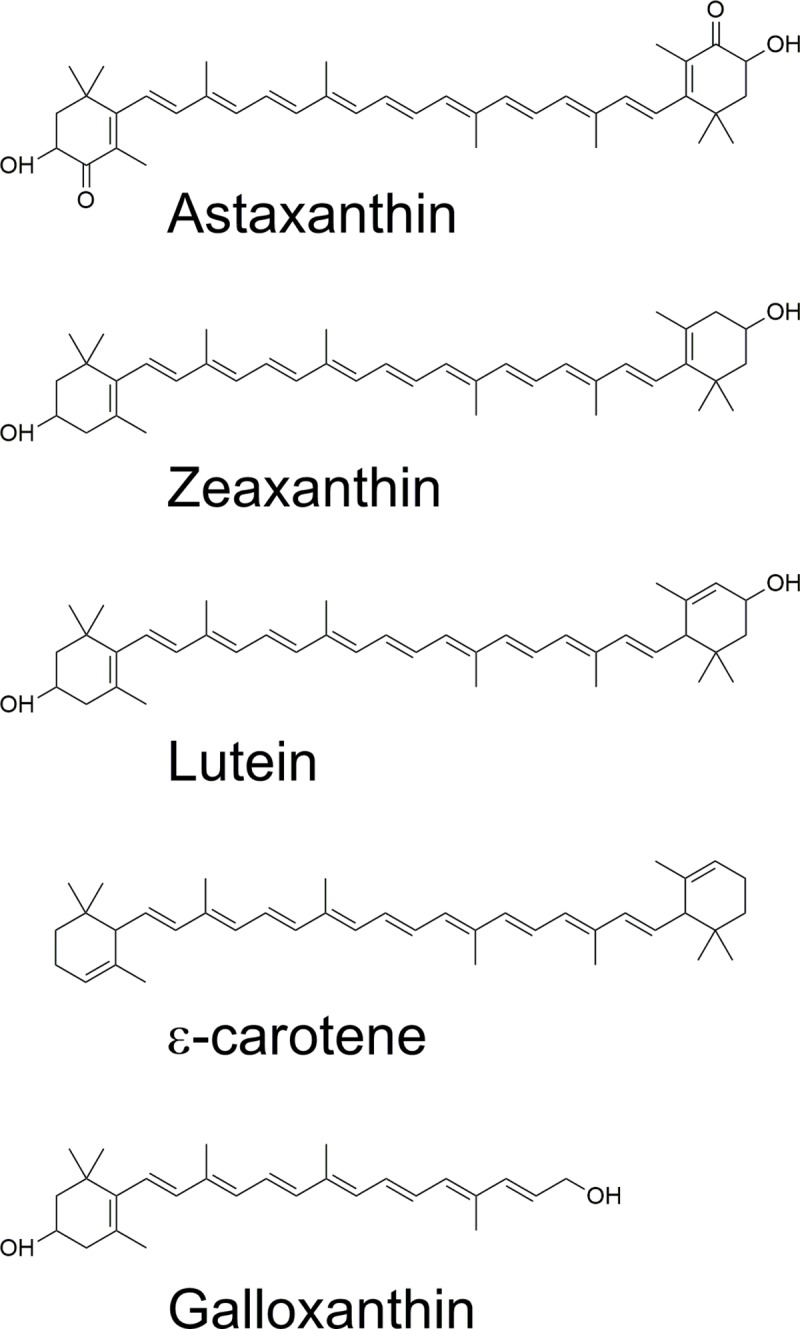
Chemical structures of the five main carotenoids found in retinas of the common chicken.

Carotenoids play a number of important roles throughout biology, and central to these functions are their electronic properties, arising from their conjugated C = C double bond chain (Fig 1). The clearest manifestation of these electronic properties is found in the colour carotenoids provide to the tissues where they are accumulated. Carotenoid absorption spectra are generally comprised of three peaks, and the lowest in energy (longest wavelength) corresponds to the main (0,0) electronic transition, the others corresponding to vibrational sub-levels of this transition. The energy of this electronic transition, which corresponds to a transition from the ground state to the second excited state [4], tightly depends on the number of conjugated carbon-carbon double bonds present in this chain. In recent years, resonance Raman spectroscopy has become a method of choice in the evaluation of the structural and functional properties of carotenoid molecules [5,6]. It can be used to identify the different carotenoid species present in a mixed population, as well as providing a detailed analysis of their molecular conformation and configuration. By matching the excitation wavelength used to produce the Raman effect to the position of an electronic transition of the scattering molecule, the Raman signal is enhanced by up to six orders of magnitude. In these *resonance* conditions, measurements can be made in complex media–*e*.*g*. on carotenoids or other pigmented cofactors bound to their protein hosts, and even *in vivo*. The resonance intensity depends directly on the relative positions of the electronic transition of the scattering molecule and the excitation wavelength used. Thus for a sample containing several pigment populations, each of which possesses slightly different absorption properties, the resonance intensity of each of the species will be slightly different for each excitation wavelength used [6]. An ensemble of Raman spectra measured at different wavelengths may thus be used to provide information on each of the species in the mixed population. Not only is this true for chemically different pigment molecules, but also for chemically identical molecules with different absorption properties (as a result of tuning by their environment). As an example, the two lutein molecules bound to the major higher plant light-harvesting complex exhibit shifted absorption bands relative to one another, and so they can be observed selectively by resonance Raman [6].

As a vibrational spectroscopy, resonance Raman provides precise molecular details of structure, interactions and environment for the molecule under study. Carotenoid resonance Raman spectra are made up of four groups of bands, termed ν_1_ to ν_4_ (see Results section). The ν_1_ band around 1500 cm^-1^, which arises from conjugated C = C stretching modes, gives direct access to the extent of the carotenoid conjugated chain. The structure of the envelope of bands around 1200 cm^-1^, termed ν_2_, is sensitive to the molecular configuration of the scattering carotenoid. The number and position of satellites observed in the ν_2_ region can therefore be used, in conjunction with the ν_1_ position, to determine the stereoisomerism of the molecule (*trans*/*cis*). Details of the carotenoid conformation may be obtained through analysis of the ν_4_ band, around 950 cm^-1^, which corresponds to out-of-plane motions of the H nuclei along the conjugated chain. For a perfectly planar carotenoid, these modes will not be coupled with the electronic transition (as the latter is oriented along the plane of the molecule) and so they exhibit no resonance enhancement—thus the intensity of this band is very weak. However, if the carotenoid is distorted out of the plane, *e*.*g*. due to steric hindrance within a protein binding pocket, then ν_4_ will gain in intensity and structure. The shape of this band can therefore yield information about the planarity of the carotenoid molecule.

Until recently, it had been assumed that each type of oil droplet in the avian retina contained only one or two carotenoid species–thus the red, yellow and colourless droplets were thought to contain astaxanthin (esters), zeaxanthin and the apo-carotenoid galloxanthin, respectively, while the pale droplets had galloxanthin and ε-carotene [7] (see Fig 1 for structures). Note that carotenoids cannot generally be synthesised by species in the animal kingdom (with a few rare exceptions among arthropods [8,9]), obtaining them instead through their diet—in some cases modifying them before their deposition in the appropriate tissue(s) [10]. Zeaxanthin and lutein (see below) are readily available in avian diets, but the other carotenoids present result from their enzymatic conversions [11,12].

More recent modelling of the absorption properties of chicken oil droplets, as measured by state-of-the-art microspectrophotometry, has led Toomey *et al*. [13] to deduce a more complex picture, in which all carotenoid-containing droplets contain a mixture of all the retinal carotenoids present–with the precise ratio of this mixture determining droplet colour and type. While spectral fitting cannot be regarded as definitive, the significant improvement in quality of the measured absorption spectra lends weight to their conclusions. In addition, Toomey *et al*. observed the presence of additional carotenoid species that had not previously been identified as components of the avian retina–lutein, and two apo-carotenoids of shorter conjugated chain length than galloxanthin (one shorter apo-carotenoid had been suggested previously [7]). On the other hand, relatively low amounts (~3%) of -carotene were observed [13], in contrast to previous studies where it was supposed to play a more significant role [7].

Here we present an investigation of the carotenoids in retinal oil droplets of the domestic chicken, *Gallus gallus domesticus*, by confocal resonance Raman microscopy. Using a range of excitations in the blue-green, we identify the carotenoids present in each type of oil droplet unambiguously, and discuss the significance of their observed conformation.

## Materials and methods

### Ethics statement

Live white and brown leghorn chickens (*Gallus gallus domesticus*, 20–23 days old) were euthanized with carbon dioxide asphyxiation, in compliance with the European Directives for the processing of animal tissues and cells. All experiments were in accordance with approval DC-2008-346 from the French Ministry for Higher Education and Research, and this study was specifically approved by the Charles Darwin Ethical Committee for Animal Experimentation.

### Sample preparation

All solvents were purchased from Sigma-Aldrich (St Louis, MO). Isolated lutein and zeaxanthin were obtained as described previously [14]. Astaxanthin and -carotene were the kind gift of Prof. Hideki Hashimoto; the latter was further purified by thin-layer chromatography, using hexane as mobile phase. The galloxanthin fraction from HPLC of chicken retinas (see below) was further purified using a YMC Carotenoid column (5 μm particle size, 4.6 x 250 mm) with an isocratic delivery of acetonitrile. For retinal samples, the eyes were isolated from the head of euthanized chickens. After incision, the anterior segment was cut off with scissors. The iris, lens, ciliary body and anterior sclera were dissected out, and most of the vitreous removed. The central retina was trepanned with a 10 mm circular punch, separated from the underlying retinal pigment epithelium and overlying vitreous, and placed on glass microscope slides for use in confocal Raman measurements. The slides were viewed under an epi-fluorescence microscope using visible and UV illumination (Leica DM5000, Germany).

### Carotenoid analysis

Retinal tissue was ground for 2 min in a 15 mL glass tissue homogenizer (Wheaton), in small volumes of ice-cold phosphate-buffered saline in the presence of 3 mL hexane. Extracted samples were placed on ice to facilitate phase separation, and the yellow-pigmented hyperphase was collected and dried under a gentle stream of nitrogen. Dried pigments were kept at -20°C in the dark prior to further analysis.

Absorption spectra of the whole pigment extract in ethanol were recorded using a Varian Cary 50 UV/Vis spectrophotometer. The samples were then split into three, with two aliquots subjected to saponification using 0.02 M or 0.2 M NaOH, to cleave ester linkages of keto-carotenoids and xanthophylls, respectively–as described elsewhere [15]; the third aliquot was run on the HPLC system without saponification. Samples were dried under a gentle stream of nitrogen gas, dissolved in 1 mL of acetonitrile/methanol/water, 87:10:3 (v/v/v), filtered with a syringe filter, and injected into a Waters 600E/600S multi-solvent-delivery HPLC system equipped with a Waters 2996 photodiode array detector. The protocol employed a Waters NovaPak C18 column (4 μm particle size, 3.9 x 300 mm), with the mobile phase programmed as follows: 0–15 min, isocratic delivery of 99% acetonitrile/methanol/water, 87:10:3 (v/v/v) and 1% ethyl acetate; 15–40 min, linear gradient to 60% acetonitrile/methanol/water, 87:10:3 (v/v/v) and 40% ethyl acetate; and 40–60 min, isocratic delivery of 60% acetonitrile/methanol/water 87:10:3 (v/v/v) and 40% ethyl acetate. The flow rate was 1.0 mL/min. Chromatogram peaks were identified by their absorption spectra and retention times, in comparison with the purified standards described above. A typical HPLC trace is presented in S1 Fig.

### Spectroscopic measurements

Resonance Raman spectra were measured using a Jobin Yvon U1000 double-grating spectrophotometer (1800 grooves/mm; entry slit 200 μm), equipped with a front-illuminated, deep-depleted CCD detector (Synapse Horiba, Jobin Yvon, Longjumeau, France). For macroscopic measurements on carotenoids *in vitro*, the signal was collected at 90° geometry. A confocal microscope with 10X objective was coupled to the Raman spectrometer for the analysis of retinal tissue (back-scattering geometry); the sample was maintained at ~ 110 K in a nitrogen-flow cryostat (Hires). Excitation wavelengths at 457.9, 488.0 & 514.5 nm, and at 413.1 nm, were provided by Innova argon and krypton lasers, respectively (Coherent, Palo Alto, USA), and at 441.6 nm by a helium-cadmium laser (Liconix). Low intensity laser power was used to prevent degradation of the sample by the absorbed light energy (less than 20 mW for macroscopic measurements; 20 μW through the microscope); acquisition times were typically 100–600 s. Sample integrity was ensured by a systematic comparison of the spectra throughout the time duration of each experiment. Note that the T-type droplets exhibited no measurable carotenoid Raman signal at any of the excitation wavelengths used.

## Results

### Oil droplets of the chicken retina

**Fig 2:**
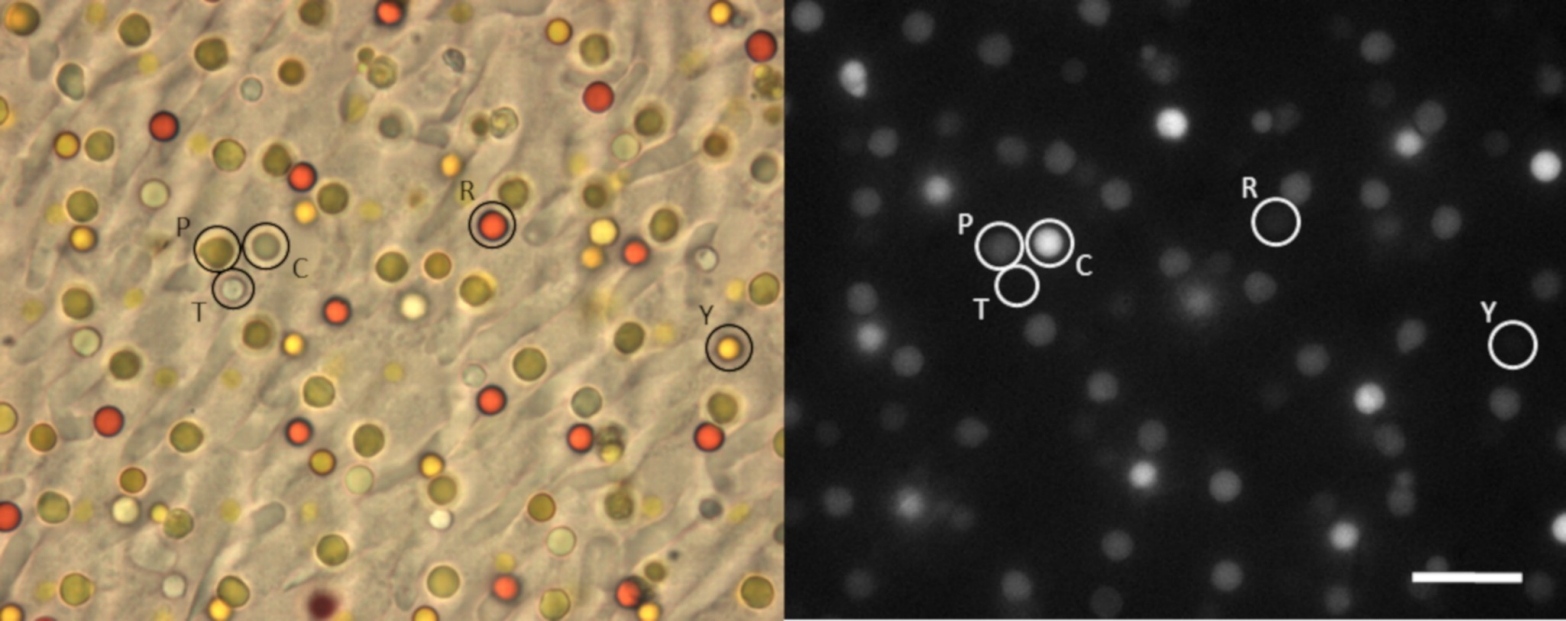
Light microscope images of the chicken retinas measured in this study. Samples were viewed under visible and UV illumination (left, right respectively). Examples of each droplet type are indicated (P—pale, R—red, Y—yellow, C–clear, T–transparent; scale bar = 10 μm).

The retinal oil droplets of the domestic chicken, *Gallus gallus domesticus*, are easily distinguished under the microscope (Fig 2). They have been classified into five types, based on their appearance and on microspectrophotometer measurements [7,13,16,17]:

**P-type (pale yellow or green); (0,0) transitions at 420, 460 nm**—principal oil droplet, and the largest (3.5–4.5 μm), associated with principal member of double cones (LWS opsin, λ_max_ ~569 nm). Medium fluorescence under UV illumination.

**R-type (red); 520 nm**—3–3.5 μm diameter, associated with LWS single cones (λ_max_ ~569 nm).

**Y-type (yellow); 480 nm**—3–3.5 μm diameter, associated with MWS single cones (λ_max_ ~507 nm).

**C-type (colourless); 420 nm**—2–3 μm diameter, associated with SWS2 single cones (λ_max_ ~453 nm). Strongly fluorescent under UV illumination.

**T-type (transparent)**—smallest oil droplet (1–2 μm), associated with SWS1 single cones (λ_max_ ~418 nm). No carotenoid, no significant absorbance above 320 nm; no observable fluorescence under UV illumination.

Note that under visible light, the P-, C- and T-type droplets are often difficult to distinguish, appearing white or slightly greenish depending on the focussing level. They are more easily distinguishable by their relative brightness under UV illumination (Fig 2, right panel).

### Carotenoids present in chicken retina and their resonance Raman spectra

Until recently, the composition of retinal oil droplets in chickens was thought to be fairly simple. The R, Y and C droplets were reported as containing only astaxanthin, zeaxanthin and galloxanthin, respectively, with the P-type containing galloxanthin and ε-carotene [7]. However, it should be noted that extraction and analysis of the carotenoids present has only been performed for whole retinas, and attributions have been based on comparing the absorption spectra of these carotenoids with microspectrophotometry of the droplets *in situ*. More recently, results from imaging spectroscopy were modelled in terms of multiple carotenoid species in each droplet type, with some new carotenoids identified (including lutein) [13]. HPLC analysis of isolated retinas from *Gallus gallus domesticus* confirmed the presence of astaxanthin, zeaxanthin, lutein, ε-carotene and galloxanthin (Table 1)—their structure is given in Fig 1. The results are, overall, very similar to those reported recently—in particular, we observed two additional components at similar retention times as the apo-carotenoids “apo1&2” described by Toomey *et al*. [13]. Note that one of these apo-carotenoids has recently been identified as di-hydro-galloxanthin [11]. On the other hand, we observed a significantly higher proportion of ε-carotene in our samples than that reported by Toomey et al.—~7%, as compared to 3%.

**Table 1:** Retinal carotenoid composition in the domestic chicken, *Gallus gallus domesticus*.

	% of total (± S.D.)
apocarotenoids	43 (± 1)
of which galloxanthin	28 (± 0.5)
astaxanthin (including *cis*-)	25 (± 2)
lutein	11 (± 2)
zeaxanthin	14 (± 2)
ε-carotene	7 (± 1)

Determination was by HPLC. Note that the standard deviation between the two breeds measured here (white and brown leghorn) was the same as that within each breed.

**Fig 3:**
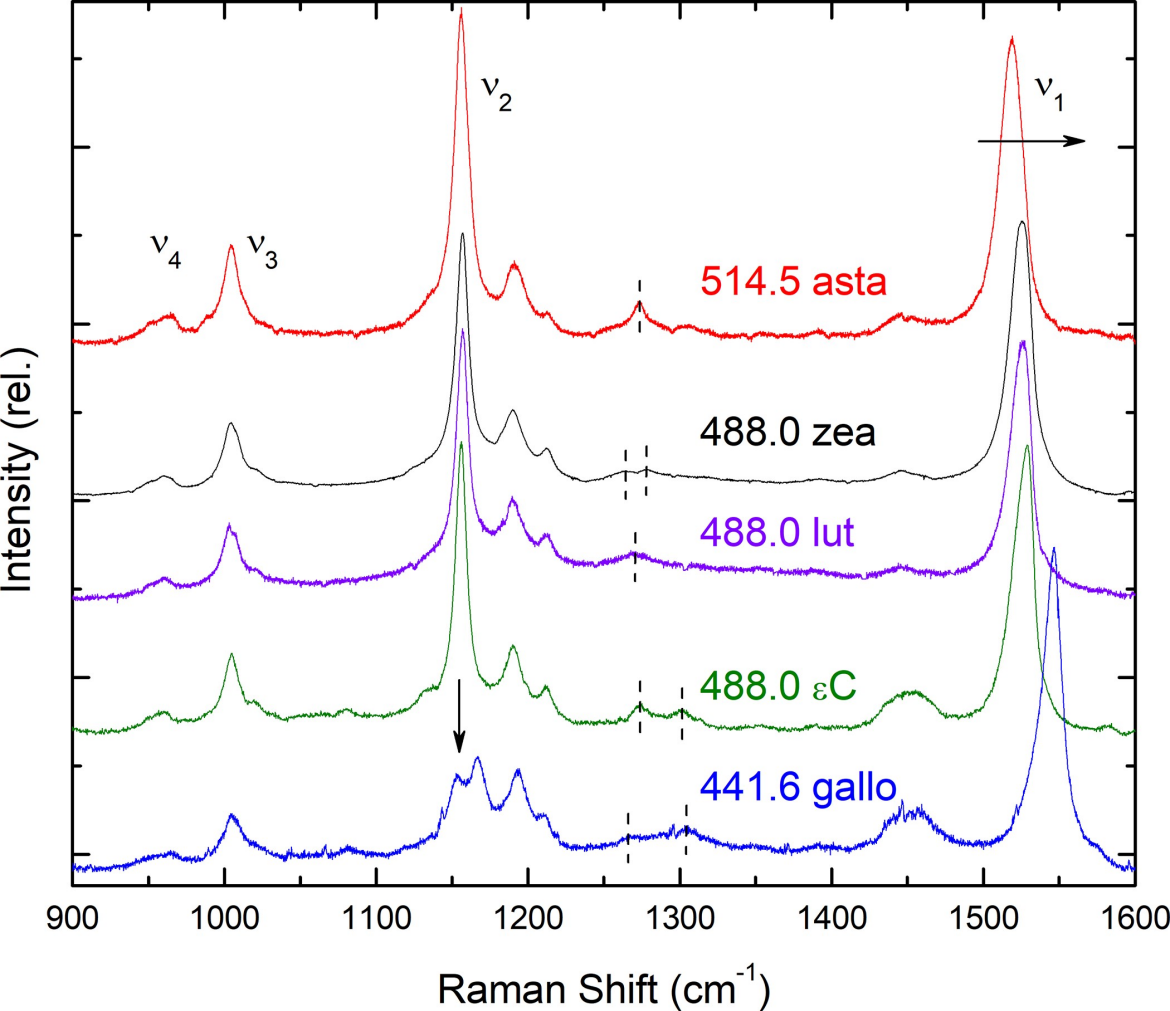
Resonance Raman spectra of avian retinal carotenoids. Spectra are shown at room temperature in the 900–1600 cm^-1^ region, for the carotenoids in Fig 1, dissolved in n-hexane. The excitation wavelength used is indicated in each case in nm; asta–astaxanthin, zea–zeaxanthin, lut–lutein, εC– ε-carotene; gallo—galloxanthin. Differences between the spectra discussed in the text are indicated by dashed lines and arrows.

Following the carotenoid analysis, the resonance Raman spectra of each isolated (unesterified) carotenoid was measured at room temperature in *n*-hexane (Fig 3). Note that ester groups on the (non-conjugated) ends of the molecule will not exert sufficient constraints to perturb the conjugated chain, and so will not affect the resonance Raman spectra–as observed for a number of astaxanthin esters [18]. The principal difference observed in these spectra is in the progression in frequency of their main ν_1_ band (horizontal arrow in Fig 3), as it directly correlates with the effective conjugated chain length N_eff_ of these molecules. Note that the position of the (0,0) absorption transition in *n*-hexane also follows this trend [5]. Thus astaxanthin (N_eff_ ~ 10.5), zeaxanthin (9.6), lutein (9.3), ε-carotene (9) and galloxanthin (7.2) exhibit ν_1_ frequencies at 1519, 1525.5, 1526.5, 1528.5 & 1546.5 cm^-1^, respectively. Further differences can be observed in and around the ν_2_ region. The full-length carotenoids are characterised by a major ν_2_ mode around 1156 cm^-1^, accompanied by two or three smaller satellite bands; this main central mode is significantly less intense for the apo-carotenoid galloxanthin (downward arrow in Fig 3)—three bands of similar intensity are observed at 1153, 1167 and 1193 cm^-1^, plus a shoulder at 1211 cm^-1^. Finally, smaller differences are seen for all carotenoids in the position and intensity of bands around 1275 cm^-1^ (dashed lines in Fig 3).

### Principal carotenoids in R and Y droplets

**Fig 4:**
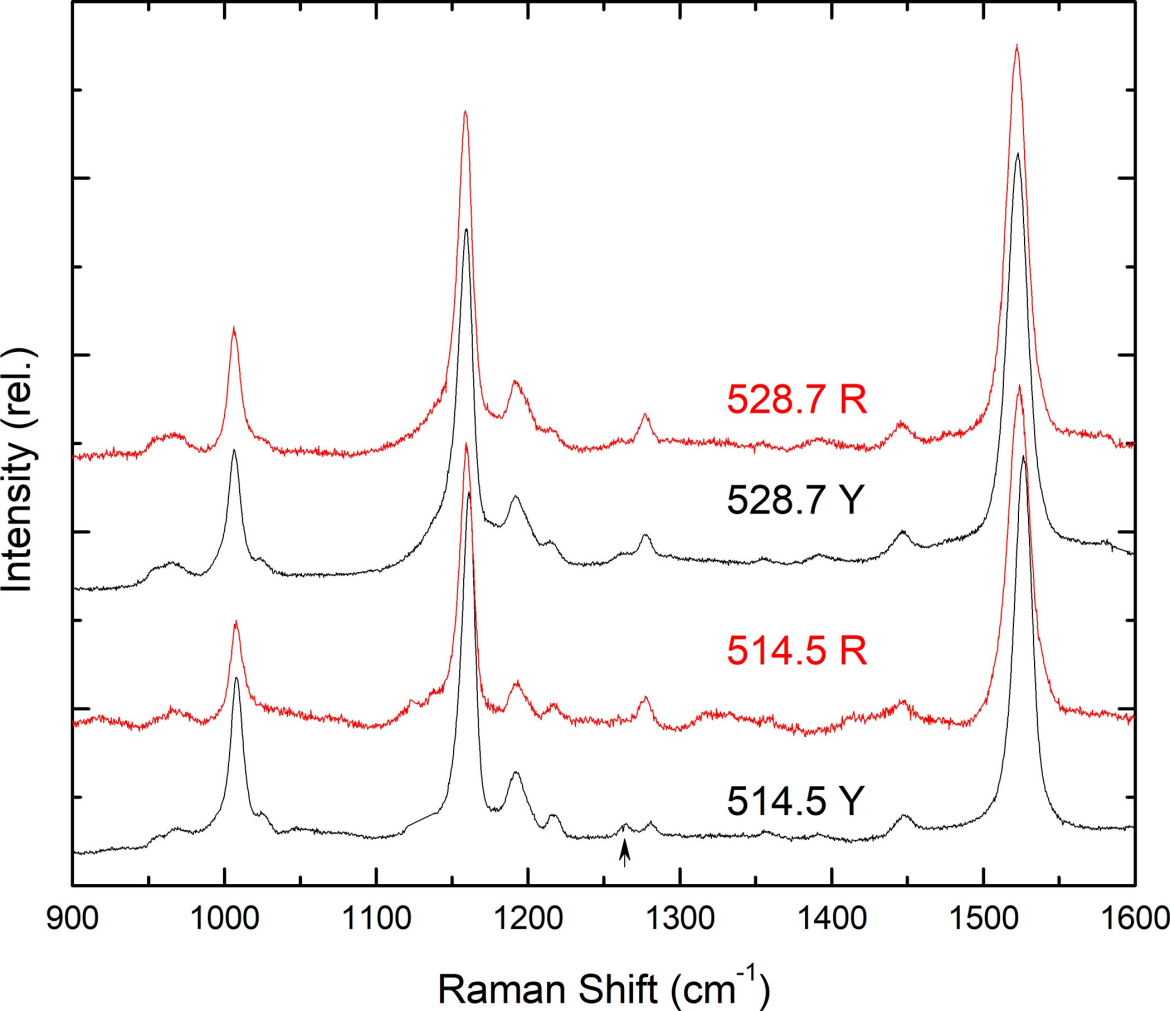
Resonance Raman spectra of R & Y droplets at 110 K, excited at 528.7 & 514.5 nm.

The main carotenoid in R droplets exhibits an apparent S_0_-S_2_ transition around 520 nm [7,13]. Resonance Raman spectra of these droplets were thus performed with 528.7 nm excitation (Fig 4). Such excitation yields spectra that are very similar to isolated astaxanthin, and the ν_1_ bandwidth (~14.5 cm^-1^) is consistent with a single carotenoid contribution at this excitation. Note that the ν_1_ bandwidth for a single carotenoid species is generally on the order of 13–15 cm^-1^, with larger values indicating the presence of more than one species. These experiments thus seem to confirm that astaxanthin is the main carotenoid in these droplets. However, when the Y droplets (which exhibit an electronic transition at 480 nm; [7,13]) are excited in the same conditions, again spectra similar to astaxanthin can be obtained, although the bandwidth of the ν_1_ mode is somewhat larger (~16.5 cm^-1^). Thus astaxanthin is in fact present in Y droplets, but the increase in ν_1_ bandwidth indicates additional contributions from other carotenoid species–indeed, it is clearly documented that the principal carotenoid in Y-droplets is not astaxanthin. It also illustrates how, in the presence of a carotenoid mixture, it is possible to observe a minor sub-population of the mixture selectively by resonance Raman spectroscopy, and that many excitation wavelengths should be used to obtain a complete picture of the carotenoid composition of the sample [6].

Exciting the R and Y droplets at 514.5 nm again yields spectra in which the position of the v_1_ is very similar, although higher than at 528.7 nm (1523.8 & 1526.2 cm^-1^ for R & Y, respectively). Note, however, that in the red droplets, this band broadens (FWHM ~ 16 cm^-1^; Fig 4)—indicating contributions of more than one carotenoid population–while for the yellow droplets, it narrows significantly to ~13 cm^-1^, so that only one carotenoid population is contributing to the latter spectrum. In addition, the features around 1275 cm^-1^ in these spectra differ significantly between R and Y droplets—the 1277.5 cm^-1^ band in red droplets is significantly larger than that at 1264 cm^-1^, as observed in spectra of isolated astaxanthin, while in Y droplets these two modes are similar in intensity, as is the case for isolated zeaxanthin (see upward arrow in Fig 4). These observations indicate that zeaxanthin is the main carotenoid in Y droplets, but that it is also present as a minor contribution in R droplets.

**Fig 5:**
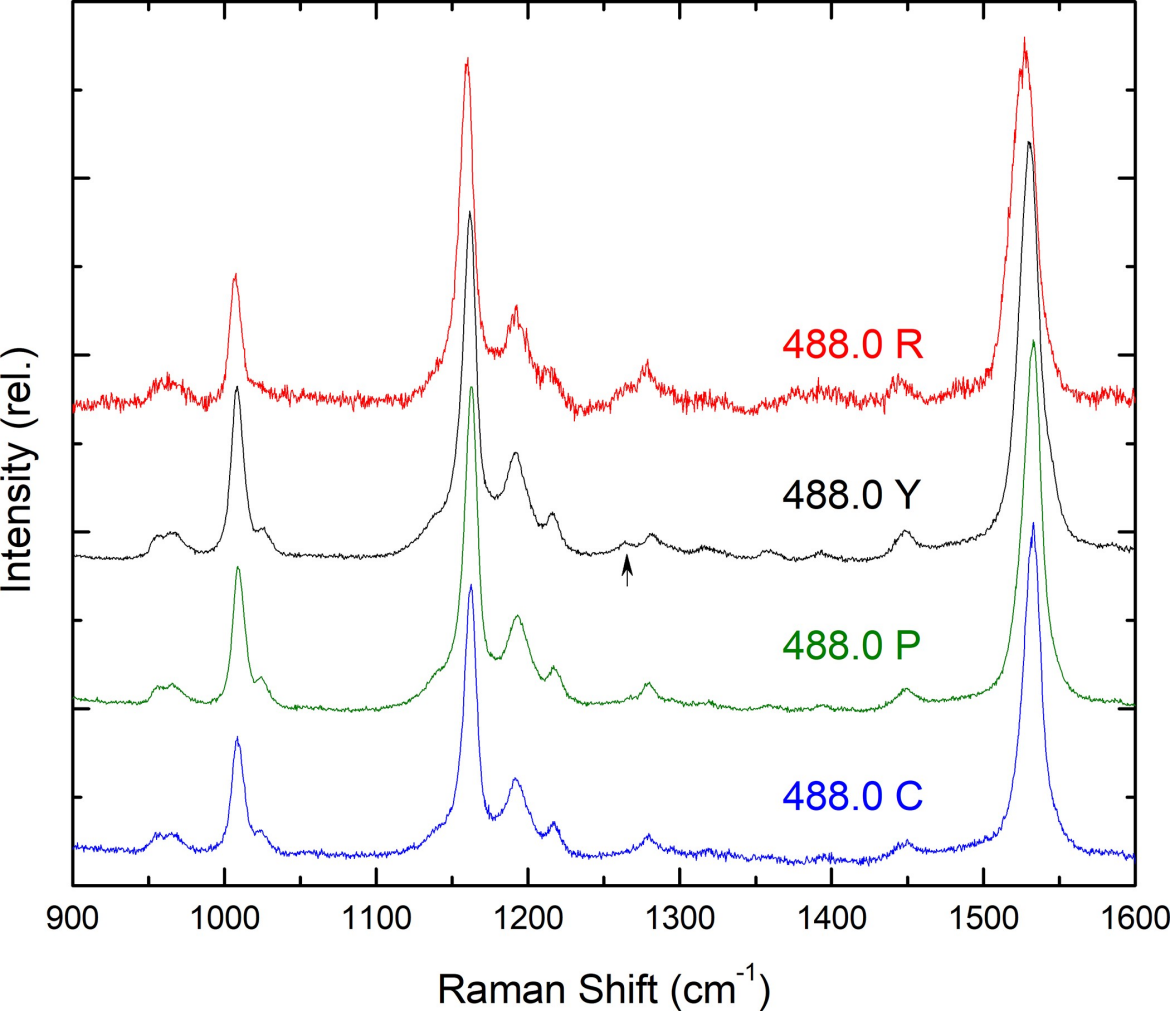
Resonance Raman spectra of all droplet types at 110 K, excited at 488.0 nm.

Presented in Fig 5 are the resonance Raman spectra of these two droplet types excited at 488.0 nm. In both spectra, a significant up-shift in the ν_1_ frequency is observed to 1527.3 & 1530.4 cm^-1^ (R, Y respectively). The bandwidth of this mode is even larger for the R-droplets than at longer wavelengths (~18 cm^-1^), while for the Y-type droplets it is intermediate in width at ~15 cm^-1^. This trend in v_1_ frequency is consistent with the presence of significant lutein contributions at this excitation, for both droplet types. Analysis of the two ~1275 cm^-1^ modes tends to confirm this, where both R- and Y-droplets exhibit features intermediate between their respective bandshapes when excited at 528.7 and 514.5 nm (note that the spectrum for lutein in this region is intermediate in shape between astaxanthin and zeaxanthin; Fig 3).

Finally when analysing the ν_4_ region of the spectra, these out-of-plane modes are clearly enhanced in both R- and Y-droplets as compared to the isolated carotenoids, for all excitations measured. This indicates that the majority, at least, of carotenoid molecules present are in a somewhat constricted environment, consistent with their binding to a specific locus.

### Principal carotenoids in C and P droplets

**Fig 6:**
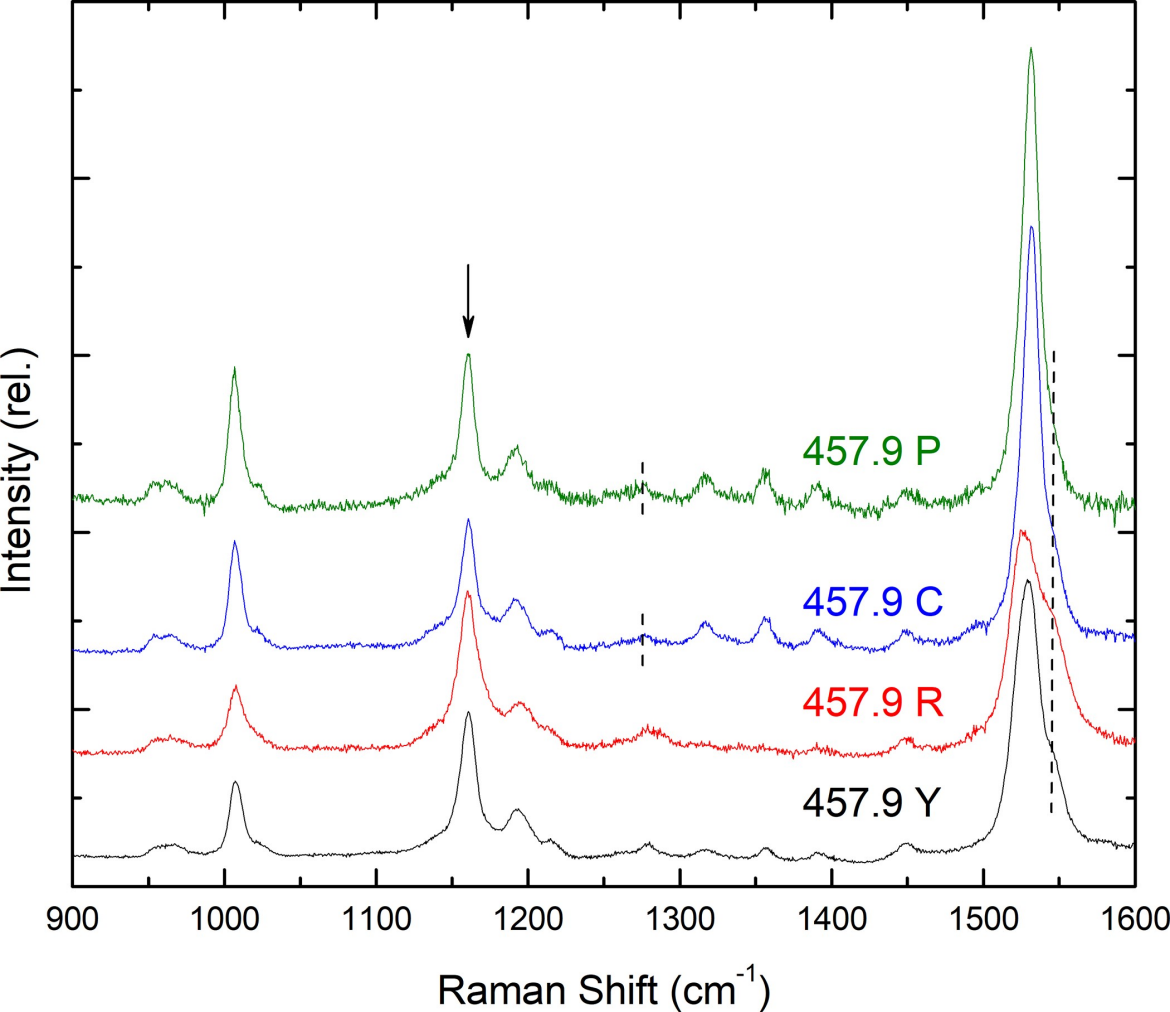
Resonance Raman spectra of all droplet types at 110 K, excited at 457.9 nm.

C- & P-type oil droplets contain carotenoid(s) whose apparent S_0_-S_2_ transition is around 420 nm, while the P-type have at least one additional carotenoid with S_0_-S_2_ transition around 460 nm. Following the same approach as for the R and Y droplets, resonance Raman spectra of these two droplet types excited at 457.9 nm were measured and are presented in Fig 6. Again, almost identical spectra for both droplet types are observed at this wavelength. Comparing the ν_1_ frequency and the shape of the ν_2_ region to those of the isolated carotenoids, it clearly appears that the major spectral component corresponds to ε-carotene–note the ν_1_ frequency around 1531.5 cm^-1^, and the band at 1277 cm^-1^ for both droplet types (dashed lines in Fig 6). The major difference in the spectra is seen in the size of the observed shoulder on the higher frequency side of the ν_1_ band, around 1546 cm^-1^ (see long dashed line in Fig 6)—this shoulder is clearly more intense for the C-type droplets. As this frequency corresponds to the expected value for galloxanthin (see Fig 3) this is a clear indication that, as for the R & Y pair of droplets, the C- & P-type contain different relative amounts of the same carotenoids. Note also the reduced intensity of the main ν_2_ mode, clearly indicating the presence of galloxanthin contributions (downward arrow in Fig 6; *c*.*f*. Fig 3). Comparing the relative intensities of the 1546 cm^-1^ shoulder, it appears that the C-droplets contain more galloxanthin than the P-type relative to ε-carotene. The contribution of galloxanthin molecules is relatively small in both cases, as the excitation line lies on top of the ε-carotene peak, but some 40 nm to the red of the galloxanthin absorption. To observe the galloxanthin contributions more clearly, resonance Raman spectra of P and C droplets were recorded at 413.1 nm, closer to the 420 nm absorption transition of this carotenoid. This excitation was seen to induce a rapid bleaching of the carotenoids present in the droplets, and the loss of the resonance Raman signal. Although the spectra obtained at this excitation are thus of lesser quality, analysis of the ν_1_ mode confirmed that the 1546 cm^-1^ contribution was significantly increased at this wavelength, and was again more prominent in the C-type droplets (S2 Fig). Interestingly, these spectra excited at shorter wavelengths also lend support to the presence of (apo-)carotenoid species with shorter conjugation lengths than galloxanthin–in addition to the contributions from ε-carotene and galloxanthin (~1530 & 1545 cm^-1^), two to three others could be observed in the 1555–1565 cm^-1^ region (upward arrow in S2 Fig). Note that for dihydro-galloxanthin (N_eff_ ~6.3), ν_1_ is expected to appear at around 1560–1565 cm^-1^.

When considering the lower frequency region of these spectra, the ν_4_ modes are again clearly enhanced relative to those of the isolated carotenoids, indicating a constrained structure and therefore a specific binding site at least for the ε-carotene present. Note that it is not possible to affirm the same for the galloxanthin molecules, as the ν_4_ region could not be recorded with a sufficient signal-to-noise ratio at 413.1 nm.

### Minor carotenoid contributions

Until fairly recently, it was thought that each oil droplet contained at most only a subset of the carotenoids present in the chicken retina. However, more recent in-depth studies using improved microspectrophometric methods appear to show that all retinal carotenoids are in fact present in every droplet type, in varying ratios [13]. We therefore attempted to confirm this by comparing the spectra for all droplets at all excitation wavelengths. For excitations at 488 nm (Fig 5), the P- and C-type droplets exhibit spectra that are clearly very similar to those for R- & Y-droplets. Indeed, they more closely resemble red droplets than yellow ones, both in their ν_1_ value (1532.7 cm^-1^) and their shape around 1275 cm^-1^. This would suggest that both P and C contain small but significant quantities of both astaxanthin and zeaxanthin/lutein.

Similarly, when considering excitations at 457.9 nm, R and Y also show clear evidence of the shorter wavelength carotenoids (Fig 6). Both droplet types exhibit a main ν_1_ peak at ~1530 cm^-1^ (R having a second one at 1525.5 cm^-1^ of similar intensity), along with the 1546 cm^-1^ shoulder, as well as showing the higher ν_1_:ν_2_ ratio (although not as high as for P and C) consistent with galloxanthin contributions. It is of particular interest that the relative size of the 1546 cm^-1^ galloxanthin band is greater for Y, and even more so for R, than for C. This indicates that the relative proportions of galloxanthin:ε-carotene increase in the sense P<C<Y<R. Unfortunately, we were unable to obtain spectra for red or yellow droplets excited at 413.1 nm, to confirm whether the other apo-carotenoids were also present (as observed by Toomey *et al*. [13]).

## Discussion

Using confocal resonance Raman spectroscopy, we have been able to distinguish the different carotenoids in retinal oil droplets of the domestic chicken. All carotenoids present are in a constrained conformation, consistent with their binding to a specific locus. The nature of this binding locus is not immediately obvious. Carotenoids in biological tissues are generally located in defined binding pockets in specific proteins, and this binding results in structured ν_4_ profiles as observed here. However, no proteins have been identified in any biological system up to now that can be fully-embedded in a lipid phase, and so their presence in retinal oil droplets seems unlikely. It is possible that significant inter-molecular interactions occur between carotenoid molecules, particularly given the high carotenoid concentration in the droplets. Such packing interactions may result in constraints in their conformation, as previously described for inter-chromophore interactions in caroteno-porphyrin dyads [19]. However, given the mixture of carotenoids present in each droplet, and with no obvious mechanism for controlling these interactions, it is hard to imagine how they could be homogeneous enough for each carotenoid species to give the ν_4_ structure observed here. This is even more the case when comparing the spectra at a single wavelength for each droplet type– ν_4_ appears very similar for any given carotenoid, whichever droplet type it is observed in, so the constraints experienced by each carotenoid are the same irrespective of the carotenoid mixture within which it is found. It therefore appears more likely that the carotenoids are constrained by binding at some other specific locus within each oil droplet, and the only remaining possibility would be in a specific lipid molecule. Modelling of the carotenoid structures may provide further insights into the nature of this binding.

While each droplet type contains a different major carotenoid, we confirm recent conclusions that all carotenoid species are found in each of the carotenoid-containing droplets [13]. (Note that, in contrast to this previously-published work, we observe significant contributions of ε-carotene in all four droplet types–in line with the higher proportion of this carotenoid measured here.) Confirmation of the findings of Toomey *et al*. is significant, as prior to this recent analysis, it was assumed that only a subset of the carotenoids at most were present in each droplet type. The reason for this complex pattern of carotenoid distribution is not obvious–the cut-off wavelength of a droplet is principally determined by the major carotenoid present, while the presence of minor species will have little effect [13]. If the basis for these complex mixtures is not functional, it may rather reflect the regulatory mechanism which produces the range of carotenoids present. Only the lutein and zeaxanthin in the droplets are acquired by the chicken in its diet, while the others are formed through enzymatic conversions of zeaxanthin [11,12]. For any given enzyme at a given concentration, only a proportion of the substrate carotenoid will be converted, based on the affinity K_d_ of the carotenoid for the enzyme. It seems reasonable to suggest that droplets are differentiated by simply varying the relative concentrations of each of the enzymes involved, such that the required major carotenoid is produced. This would have the side-effect of producing all of the carotenoids in every droplet type, but at different relative concentrations, depending on the threshold value for each carotenoid at the given enzyme concentration.

## Supporting information

S1 Fig Typical HPLC trace of carotenoid extract from white leghorn chickens, upon saponification with 0.02 M NaOH. Detection was at 450 nm. 1 –galloxanthin; 2 –astaxanthin; 3 –*cis*-astaxnthin; 4 –lutein; 5 –zeaxanthin; 6 – ε-carotene. Shorter chain-length apo-carotenoids are indicated by arrows; additional species eluting in the 40–60 min region are carotenoid esters.
(EPS)

S2 Fig Resonance Raman spectra of P & C droplets at room temperature, excited at 413.1 nm. The ν_1_ position of galloxanthin is shown with a dotted line, while additional contributions from shorter-length carotenoids are indicated by an arrow.
(TIF)
