# Supplementary figures and images for: Carotenoid composition and conformation in retinal oil droplets of the domestic chicken*
Source: PLoS One. 2019 May 31;14(5):e0217418. doi: 10.1371/journal.pone.0217418 (PMC6544226; doi:10.1371/journal.pone.0217418)

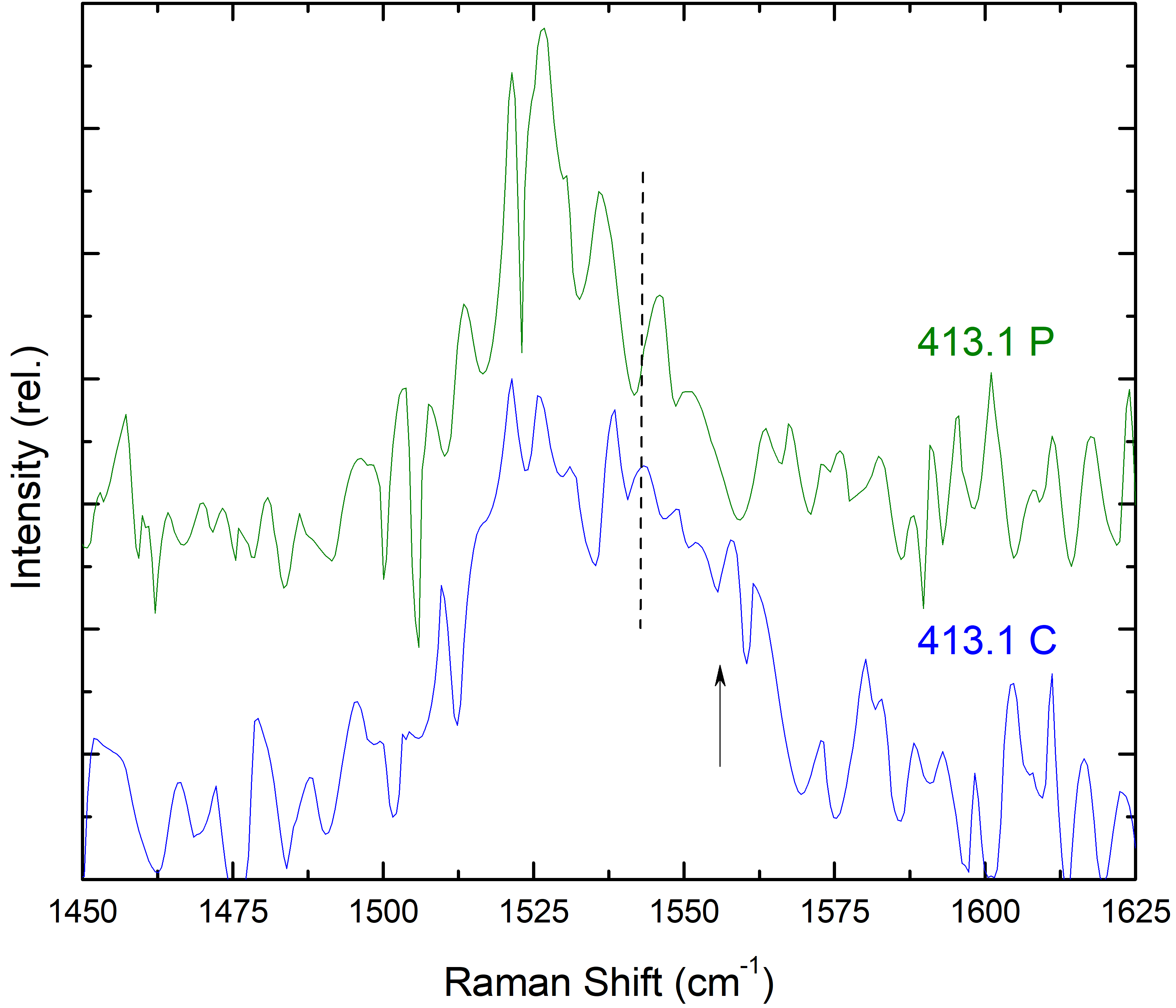

Supplement: S2 Fig — The ν1 position of galloxanthin is shown with a dotted line, while additional contributions from shorter-length carotenoids are indicated by an arrow. (TIF) [file pone.0217418.s002.tif]
